# Multi-omics analysis of epigenetic dysregulation reveals clinical heterogeneity and evaluates the immunotherapeutic potential of lung adenocarcinoma

**DOI:** 10.1016/j.gendis.2025.101561

**Published:** 2025-02-20

**Authors:** Yuqing Ren, Zaoqu Liu, Yi Yue, Siyuan Weng, Hongxia Jia, Jing Li, Ping Li, Chunya Lu, Xinwei Han, Guojun Zhang

**Affiliations:** a Department of Respiratory and Critical Care Medicine, The First Affiliated Hospital of Zhengzhou University, Zhengzhou, Henan 450052, China; b State Key Laboratory of Medical Molecular Biology, Institute of Basic Medical Sciences, Chinese Academy of Medical Sciences, Department of Pathophysiology, Peking Union Medical College, Beijing 100730, China; c Department of Interventional Radiology, The First Affiliated Hospital of Zhengzhou University, Zhengzhou, Henan 450052, China

Lung cancer is the leading cause of cancer-related mortality globally, including small-cell lung cancer and non-small-cell lung cancer. As the most prevalent histological subtype of non-small-cell lung cancer, lung adenocarcinoma (LUAD) accounts for approximately 40% of all lung cancer cases.^1^ Due to the heterogeneity of LUAD, accurate categorization is required to create a treatment plan for LUAD patients, while the existing paradigm does not adequately capture the enormously heterogeneous characteristics of LUAD. The rise of epigenetics has brought new perspectives for tumor heterogeneity exploration. Epigenetic modifications, such as aberrant DNA methylation and microRNA (miRNA), are essential in controlling gene expression, heterogeneity, and clinical implication.^2^ Meanwhile, epigenetic disruptions contribute to lung cancer tumorigenesis, the generation of a malignant phenotype and aggression, and chemoresistance, which could serve as credible biomarkers for lung cancer molecular categorization, early diagnosis, prognosis classification, and treatment efficacy prediction.^3^ Through integrative clustering of the gene expression profiles regulated by epigenetics, we determined and validated four lung adenocarcinoma epigenetic subtypes (LAESs) with distinct prognoses and biological peculiarities from four independent multi-center lung adenocarcinoma cohorts.

The overall schematic outline of our study was performed in Figure S1. Negatively correlated gene signatures were identified for promoter DNA methylation correlated genes (METcor genes, *n* = 1053; Table S1) and miRNA expression associated genes (MIRcor genes, *n* = 680; Table S2), and only 36 genes were found to overlap (Fig. S2B), indicating that DNA methylation and miRNA are less possibly to co-regulate the same gene. METcor genes were found in opensea regions and the 5′UTR rather than other sites, which implies that methylation in the opensea regions and/or 5′UTR locations may play a role in mRNA regulation (Fig. S2C). Furthermore, pathway analyses revealed the difference in gene function enrichment between METcor and MIRcor genes (Fig. S2D; Table S3, 4). These findings revealed that the METcor and MIRcor genes might perform multiple biological features in regulating gene expression from different aspects. As shown in Figure 1A, the frequency of aberrations between the METcor gene and the MIRcor gene showed a high synchronous performance (*r* = 0.758, *P* < 0.001). In addition, there was a marked association between high and low expression of METcor or MIRcor genes in pairwise comparisons (Fig. 1B). Aberrant METcor and MIRcor genes appear synergistically regulated in LUAD.

**Figure 1:**
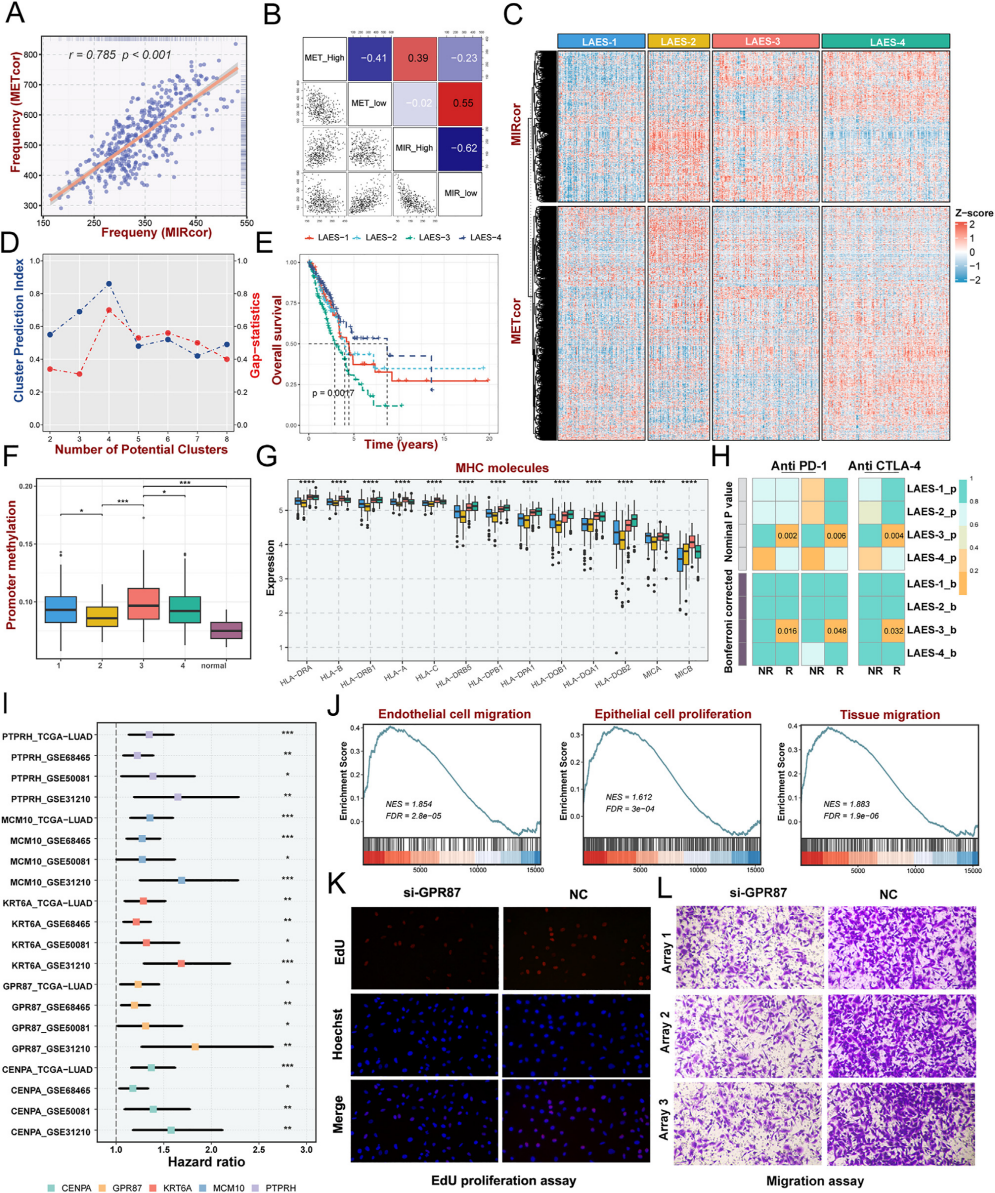
Identification and validation of four lung adenocarcinoma epigenetic subtypes with distinct prognosis and biological peculiarities. **(A)** Correlation between the frequencies of aberrant METcor and MIRcor genes in each sample of the TCGA dataset. **(B)** Pairwise correlations between the frequencies of METcor high, METcor low, MIRcor high, and MIRcor low genes. **(C)** Heatmaps of gene expression patterns of the LUAD subtypes identified by integrative clustering analysis for METcor and MIRcor data. **(D)** The predicted number of clusters by the CPI and Gap−statistics between 2 and 8 clusters. **(E)** Kaplan–Meier survival curves of overall survival for lung adenocarcinoma epigenetic subtypes (LAESs). **(F)** The levels of promoter methylation in the LUAD subtypes and adjacent normal samples. **(G)** The expression of major histocompatibility complex (MHC) molecules in LAESs. **(H)** SubMap analysis showed the similarity of gene expression profiles between the LUAD subtypes and patients from three independent datasets treated with CTLA4 and PD-1 inhibitors: GSE135222, GSE173839, and the Nathanson cohort. **(I)** Cox univariate analysis of LAES-3 signature genes in discovery and validation cohorts. **(J)** The GSVA analysis unveiled a noteworthy correlation between *GPR87* and cell migration, proliferation, and migration. **(K)** Representative photomicrographs of EdU immunofluorescence staining assay in A549 cells. **(L)** Transwell assays were performed to examine the potential migration of A549 cells. “R” represents responsive, whereas “NR” represents no responsive. ^ns^*P* > 0.05, ∗*P* < 0.05, ∗∗*P* < 0.01, ∗∗∗*P* < 0.001, ∗∗∗∗*P* < 0.0001.

Based on the premise of significant co-regulation between aberrant frequencies of MIRcor and METcor genes, we performed an integrative clustering analysis for the METcor and MIRcor gene expression profiles to establish LAESs, and four clusters were determined to be the best solution based on the CPI and Gap-statistics (Fig. 1C, D). Significant differences were demonstrated in survival among the LAESs. LAES-4, the largest subtype, was associated with the best prognosis, while LAES-3 showed the poorest prognosis (*P* = 0.0017; Fig. 1E). The prognoses of LAES-1 and LAES-2 were somewhere in between. To confirm the robustness of the clustering pattern, we predicted LUAD subtypes in three independent GEO datasets with the NTP algorithm and validated survival differences among the LAESs across distinct cohorts (Fig. S3A, B).

We then compared the accessible clinicopathologic characteristics among the four LUAD subtypes (Fig. S3E) and utilized Cox regression analyses to explore the correlation of the clinical status and each subtype with survival, which demonstrated that LAES-3 was an independent prognostic factor in LUAD (Fig. S3C). Compared with the previously published classical molecular subtype of LUAD,^4^ terminal respiratory unit patients, with the best survival rates, were highly enriched in LAES-4, accounting for 84.5%, while 81% of patients of LAES-3 significantly consisted of proximal-inflammatory type patients who had poor favorable prognoses (Fig. S3D). Proximal-proliferative type patients (70.8%) were mainly enriched in LAES-2, while patients with LAES-1 type were mainly composed of proximal-proliferative type (48.6%) and proximal-inflammatory type (32.4%).

We proceeded to analyze the patterns of DNA methylation and miRNA expression in LAESs. Through comparative analysis with other subtypes and adjacent normal samples, LAESs exhibited elevated levels of methylated CpG sites and increased miRNA expression (Fig. 1F; Fig. S4A). Additionally, the promoter hypermethylation level of LAES-3 exhibited the highest degree among the four subtypes, while the miRNA expression level of LAES-2 demonstrated the greatest intensity. To visually describe the genomic heterogeneity of LUAD patients, we explored the genomic landscape (Fig. S4B) and the top 30 frequently mutated genes (Fig. S4C) of the four subtypes. LAES-2 showed a significantly higher tumor mutation burden, single nucleotide polymorphism, and insertion-deletion (Fig. S4B, D) than the other LUAD subtypes. Further, more chromosomal aberrance was discovered in LAES-2 than in the other subtypes, with remarkably higher copy number loss or gain generally (Fig. S4E, F).

By assessing significant biological processes, we recognized individual biological features among the four subtypes (Fig. S5A–D). To show the widespread intertumoral heterogeneity of the four LUAD subtypes intuitively, a total of 55 significant biological processes of each subtype were selected to make the heatmap using ssGSEA (Fig. S6A). We found that metabolism-related pathways were up-regulated in the LAES-1. LAES-2 and LAES-3 both were characterized by elevated levels of proliferation-relative pathways. In addition, the LAES-4 exhibited moderate levels of metastasis-relative pathways and elevated cell motility-relative pathways. As expected, LAES-3 was identified by more aggressive features, containing up-regulated metastasis-relative pathways and several cancer-relative signaling pathways. To investigate the potential evolutionary changes in biological function within the four subtypes, the soft clustering method from the Mfuzz package was utilized to identify eight distinct gene clusters (Fig. S5E). Clusters 2 and 3 demonstrate a progressive alteration in gene expression patterns from the most favorable prognostic subtype LAES-4 to the least favorable prognostic subtype LAES-3 (Fig. S5E). As the prognosis deteriorated, there was a decrease in the expression of genes associated with metabolic processes (Fig. S5F), and an increase in the expression of genes related to immune response, cytokine response, and cell activation (Fig. S5G).

A more comprehensive examination of the expression profiles of immune-related genes within each subtype is conducted to predict their immune signatures and investigate the influence on the advancement and treatment responses of lung adenocarcinoma. As expected, LAES-3 performed a higher profusion of immune cell infiltration (Fig. S7A). Major histocompatibility complex molecules and the antigen processing and presenting machinery score^5^ were explored to characterize the antigen processing and presenting capacity in tumors, revealing significantly elevated levels in LAES-3 compared with other subtypes (Fig. 1G; Fig. S7C). Although the prevalence of major effector cells involved in anti-tumor immunity was notably greater in LAES-3 compared with the other subgroups, it expressed more elevated levels of immune-excluded contents, and suppressive immune genes, especially *CD274*, *CTLA4*, and *PDCD1* (Fig. S7B, E). Based on these results, patients in LAES-3 may benefit from immunotherapy. As expected, LAES-3 was positively linked to most immune cells and cancer–immunity cycle steps, and it had a high level in the score of each step (Fig. S7F, G). As depicted in Figure S7D, LAES-3 conveyed the highest TIS score, indicating that patients in LAES-3 were more likely to benefit from immunotherapy. Meanwhile, LAES-3 shared a similar gene expression profile with anti-PD-1 and CTLA4 responders from three immunotherapy cohorts, indicating that LAES-3 may benefit from anti-PD-1 and CTLA4 therapy (Fig. 1H), which provided further evidence supporting the conjecture.

To recognize signature prognosis-related genes, univariate Cox analysis and receiver operating characteristics statistics were executed for LAES-3 signature genes in four independent multi-center lung adenocarcinoma cohorts. *GPR87* was identified as the optimal prognostic characteristic gene for LAESs (Fig. 1I; Fig. S8A–C). The GSVA analysis revealed a significant association between *GPR87* and cell migration, proliferation, and migration (Fig. 1J). To investigate the effect of *GPR87* in LUAD cells, cell functional assays were performed *in vitro* (Fig. 1K, L; Fig. S8D–I). We demonstrated that silencing *GPR87* attenuated the migration and invasion of LUAD cells through wound healing and transwell assays and declined LUAD cell proliferation in CCK-8 and EdU assays. Therefore, *GPR87* has been deemed a carcinogenic factor in LUAD, enhancing the capacity of proliferation, migration, and invasion, which confers a worse prognosis in LAES-3.

In conclusion, based on epigenetically regulated gene expression profiles, four well-characterized LAESs with distinct clinical outcomes, multi-omic landscapes, biological mechanisms, and immunological features were developed and validated. Particularly, LAES-3 was considered an immune-infiltrated subtype with the worst prognosis, characterized by a high *TP53* mutation burden, up-regulated proliferation, and metastasis features. This particular subtype exhibits responsiveness to immunotherapy, and the precise identification of patients with LAES-3 and subsequent immunotherapy intervention has the potential to enhance the prognosis of lung adenocarcinoma. This classification accurately stratifies patient survival risk and holds great potential for facilitating the development of clinical precision therapy in lung adenocarcinoma.

## Funding

This study was supported by Henan Provincial Key Laboratory of Medicine and Henan Provincial Clinical Medical Research Center for Respiratory Diseases.

## CRediT authorship contribution statement

**Yuqing Ren:** Data curation, Formal analysis, Methodology, Validation, Writing – original draft. **Zaoqu Liu:** Formal analysis, Validation, Writing – review & editing. **Yi Yue:** Writing – review & editing. **Siyuan Weng:** Writing – review & editing. **Hongxia Jia:** Writing – review & editing. **Jing Li:** Writing – review & editing. **Ping Li:** Writing – review & editing. **Chunya Lu:** Writing – review & editing. **Xinwei Han:** Writing – review & editing. **Guojun Zhang:** Writing – review & editing.

## Data availability

Public data used in this work can be acquired from the UCSC Xena website (https://xena.ucsc.edu/), TCGA database (http://cancergenome.nih.gov/), and Gene Expression Omnibus (GEO, http://www.ncbi.nlm.nih.gov/geo/). The datasets presented in this study can be found in online repositories. The names of the repository/repositories and accession number(s) can be found in the article. Other data supporting the findings of this study are available from the corresponding author upon reasonable request.

## Conflict of interests

The authors have no conflict of interests to disclose.
