# Supplementary material for: Multi-omics analysis of epigenetic dysregulation reveals clinical heterogeneity and evaluates the immunotherapeutic potential of lung adenocarcinoma
Source: Genes Dis. 2025 Feb 20;12(5):101561. doi: 10.1016/j.gendis.2025.101561 (PMC12166690; doi:10.1016/j.gendis.2025.101561)
Supplement: Multimedia component 1 [file mmc1.docx]

**Supplementary Methods**

**Data retrieval and preprocessing**

Transcriptome profiling data (mRNA-Seq) and available clinical information of TCGA-LUAD patients with primary curative resection were acquired from the UCSC Xena website (<https://xena.ucsc.edu/>). After disqualified samples were removed, 435 patients with complete prognostic information were chosen for further analysis. The inclusion criteria validation cohorts were as follows: (1) the dataset must contain more than 100 LUAD patient samples, (2) it must include complete survival data for each sample, (3) each sample must have a unique sample ID to enable deduplication, and (4) the dataset must provide clear pathological classification of lung cancer to ensure accurate identification of LUAD samples. Three independent datasets as external validation cohorts were collected, including GSE68465 (*n* = 443), GSE31210 (*n* = 226), GSE50081 (*n* = 127) from the Gene Expression Omnibus (GEO, https://www.ncbi.nlm.nih.gov/geo/). To ensure that no duplicate samples were included in the analysis, we applied a sample deduplication strategy based on unique sample identifiers (IDs). In cases where multiple samples from the same patient were available (e.g., multiple tissue types or multiple time points), only the sample with the most complete clinical and transcriptomic data was retained. If any sample IDs were duplicated within the same dataset, we removed the duplicates to ensure each patient contributed only one sample to the final analysis.

DNA methylation data, miRNA expression data (miRNA-Seq), somatic mutation (MAF files), and somatic copy number variation (CNV) of patients with lung adenocarcinoma were retrieved from TCGA database (http://cancergenome.nih.gov/). Expression levels of mRNA, miRNA and CpG sites were transformed by log2 (normalized TPM+1). After normalization of the DNA methylation probes, we retained only CpG sites located in the promoter region on the non-sex chromosomes for analysis, including TSS1500, TSS200, 50UTR, and 1stExon, using the R ‘ChAMPdata’ package. The R ‘maftools’ package was used to explore somatic variants data.

**Identification of METcor and MIRcor genes**

We retrieved 81,902 CpG-mRNA pairs located in the promoter region of the mRNA based on mRNA expression and methylated CpG sites in LUAD samples from the TCGA database. To ensure that the data were not missed, we generated 36,196 pairs of reactive miRNAs and mRNAs using the three databases, including miRTarBase, miRDB, and TargetScan database^1-3^. Then, normalized Pearson correlation coefficients by Fisher Z-transformation were calculated for CpG-mRNA pairs and miRNA-mRNA pairs separately. As **Fig. S2A** shows, there was a negative correlation between CpG and miRNA interactions with mRNA (*P* < 0.001, Z-test). Only negative correlations of CpG-mRNA pairs and miRNA–mRNA pairs were chosen for downstream analysis according to the judgment that the DNA methylation pattern and miRNA are critical negative epigenetic regulators of gene expression^4,5^. Subsequently, 1,697 METcor and MIRcor genes were recognized using the Fisher Z-transformed correlation coefficients with negative 95% confidence intervals.

**Clustering analyses and validation of lung adenocarcinoma epigenetic subtypes**

Integrative clustering analysis was used for 1,687 METcor and MIRcor gene expression profiles to structure LUAD subtypes from TCGA dataset by the iClusterBayes method, implemented in R ‘iClusterPlus’ package to analyze multi-omics data. The first-rank number of clusters was confirmed by the cluster prediction index (CPI) and Gap-statistics between 2 and 8 clusters.

Differential expression analyses were conducted among each tumor subtype and contiguous normal samples using the R ‘limma’ package to identify the signature genes of each subtype in the TCGA database. The genes that differentially expressed both between normal and tumor samples (log2|fold change| > 1 and *P* < 0.05), as well as between each subtype and other tumor samples (log2|fold change| > 0.5 and *P* < 0.05) were selected to comprise the signatures (**Table S5**). Then to validate the robustness of the LUAD subtypes constructed from the METcor and MIRcor genes, we used the nearest template prediction (NTP) algorithm^6^ to identify LUAD subtypes in GEO database by signature genes for the subsequent validation.

**Pathway enrichment analysis**

A total of 9,570 gene sets (7,350 from GO biological process, 186 from KEGG, 289 from Biocarta, 1,499 from Reactome, 196 from PID and 50 from cancer Hallmark) from the MSigDB database (https://www.gsea-msigdb.org/gsea/msigdb/) were used for pathway enrich analysis using R ‘clusterProfiler’ package. Single-sample gene set enrichment analysis (ssGSEA) was performed using R ‘GSVA’ package on function pathways from curated gene sets of GO, KEGG, and hallmark gene sets. Metascape was utilized for the purpose of functionally characterizing the signature genes that arose from the soft clustering method known as Mfuzz^7,8^.

**Mutation landscape and CNVs in LAES**

To further investigate genomic features, the total number of mutations in DNA sequence nucleotides, and tumor mutation burden (TMB), were taken into account. Single nucleotide polymorphisms (SNPs), insertions and deletions (Indels) were also included in exploratory analyses of genomic features of each subtype. We analyzed fraction of genome alteration (FGA), fraction of genome gained (FGG), and fraction of genome lost (FGL) for LAES described as the fraction of total CNV/gain/loss bases to all bases in several subtypes. The top 15 amplified (AMP) and homozygous deletion (Homdel) chromosome segments were shown in each subtype. The top 20 genes in the overall mutation frequency were identified as frequently mutated genes (FMGs), and analyzed the distinct mutation frequency of these genes among subtypes.

**Immunological characteristics of LAES**

To estimate the abundance of immune infiltration in the tumor microenvironment in LAES, gene sets of 28 immune cells and 27 immune checkpoints retrieved from the published research were evaluated via the ssGSEA algorithm^9-12^. Immune scores and stromal scores were evaluated to analyze infiltration levels of immune cells in distinct subtypes using the ESTIMATE algorithm^13^. MHC molecules and the antigen processing and presenting machinery scores (APS)^14^, consisting of 16 antigen-presenting genes, were calculated to unfold the efficiency of antigen processing and delivery.

**Assessment of immunotherapy response in LAES**

We employed the tumor inflammation signature (TIS)^15^ scores based on 18 immune-inflammatory genes to predict the responses to PD-1 blockade and described the immune status of LUAD subtypes from multiple perspectives. Subsequently, the cancer-immunity cycle (CIC)^16^ immunogram, composed of seven steps, including cancer cell antigens, cancer antigen presentation, priming and activation of effector T cells, T cell migration to cancer tissue, infiltration of immune cells into tumors, recognition of cancer cells by the T cell, and killing of cancer cells, was built to describe dynamic immunity processes within tumors among four subtypes. SubMap analysis ^17^ was used to assess the similarity of gene expression profiles between the LUAD subtypes determined and tumor patients who accepted different immune checkpoint blockade therapy, including cytotoxic T lymphocyte-associated protein 4 (CTLA4) or programmed cell death protein-1 (PD-1) from three independent datasets (GSE135222, GSE173839 and Nathanson cohort)^18-20^

**Identification of signature prognosis-related genes**

Univariate Cox analysis was executed for LAES-3 signature genes in four independent multi-center lung adenocarcinoma cohorts. According to screening conditions of *P*<0.05 and HR>1, five prognosis-related genes, *CENPA*, *GPR87*, *KRT6A*, *MCM10* and *PTPRH*, were obtained after taking intersection in four cohort. After excluding cohorts with normal samples comprising less than 5% of the tumor samples, the expression of the five prognosis-related genes in the tumor was significantly raised compared to normal tissues revealed in TCGA-LUAD and GSE31210 cohort. Ultimately, the ROC analysis of *CENPA*, *GPR87*, *KRT6A*, *MCM10* and *PTPRH* suggests *GPR87* with the highest average ROC statistic was deemed to be the diagnostic gene for the worst prognosis. Moreover, *GPR87*-low and *GPR87*-high groups exhibited significant differences in overall survival by Kaplan-Meier survival analysis in all discovery and validation cohorts.

**Cell lines and cell culture**

LUAD A549 cell lines cultured in Dulbecco’s Modified Eagle’s medium (DMEM)/High-Glucose (Solarbio, Beijing, China) containing 10% fetal bovine serum (Cyagen, Guangzhou, China). We cultured the cells at 37 °C and 95% humidity with 5% CO2 in a standard tissue culture incubator.

**siRNA transfection and qRT-PCR**

The non-specific or GPR87-specific siRNAs (5′-TCCTGACACGCATCTTTGCT-3ʹ, RIBOBIO, Guangzhou, China) were transfected into A549 cells using jetPRIME transfection reagent (Polyplus-transfection® SA, France). Further, entire transcripts were demonstrated by quantitative real-time polymerase chain reaction (qRT-PCR).

**Wound healing assay**

Equal numbers of cells were seeded in 200 μL of DMEM supplemented with 10% FBS. Once a confluent monolayer was formed, scratches were created using a sterile 200 μL pipette tip. Subsequently, the cells were inoculated with serum-free medium. The width of the wounds was photographed at 0 and 48 hours using a light microscope.

**Transwell migration assay**

Transwell migration assays were performed using 24-micron pore size Transwell plates. A total of 2.5 × 10^4^ cells were resuspended in serum-free medium and seeded in the top chamber of the Transwell plate. The lower chamber was filled with complete medium containing 10% FBS DMEM. The cells were then incubated at 37 °C for 48 hours. Following the incubation period, the migrated cells were fixed and stained. The migrated cells were subsequently counted under a light microscope.

**Cell Counting Kit-8 Assay**

Cell proliferation was examined by Cell Counting Kit-8 (CCK-8, Dojindo, Kyushu, Japan). LUAD cells were seeded in 96-well plates for 24 h, 48 h, 72 h, and 96 h based on the manufacturer’s instructions. Then, 10 μL CCK-8 reagent was added to the plates and incubated at 37°C for 2 h. A microplate reader (BioTek, Winooski, VT, USA) was used to detect the absorbance at 450 nm.

**5-Ethynyl-20-deoxyuridine proliferation assay**

5-Ethynyl-20-deoxyuridine (EdU) proliferation assay was implemented by Cell-Light EdU Apollo®567 In Vitro Imaging Kits (RiboBio, Guangzhou, China). In each well of 96-well plates, 4 × 103 cells were incubated with 50 μM EdU for 2 h. After that, the cells were fixed in 4% formaldehyde for 30 min, neutralized excess formaldehyde by 2 mg/ml glycine, and washed in PBS, followed by permeabilizing with 0.5% Triton X-100 for 10 min. Next, the cells were dyed with Apollo solution and Hoechst 33,342 for 30 min, respectively. An inverted fluorescence microscope (Olympus, Tokyo, Japan) was used to picture and count the EdU-positive cells.

**Statistical analysis**

R software version 4.1.2 was used for all statistical analyses. The time from the date of primary diagnosis to the date of death or last contact was defined as overall survival (OS). The connections between the LUAD subtypes and survival were evaluated by Kaplan-Meier (KM) survival and multivariate Cox regression analyses. The categorical variables were analyzed by chi-square test or Fisher exact test. The Kruskal-Wallis rank-sum test was used to evaluate differences in continuous data between several groups. A two-tailed *P* value < 0.05 was considered statistical significance. All *P* values were adjusted using the Benjamini–Hochberg method.

**References**

1 Chou, C. H. *et al.* miRTarBase update 2018: a resource for experimentally validated microRNA-target interactions. *Nucleic Acids Res* **46**, D296-d302, doi:10.1093/nar/gkx1067 (2018).

2 Chen, Y. & Wang, X. miRDB: an online database for prediction of functional microRNA targets. *Nucleic Acids Res* **48**, D127-d131, doi:10.1093/nar/gkz757 (2020).

3 Agarwal, V., Bell, G. W., Nam, J. W. & Bartel, D. P. Predicting effective microRNA target sites in mammalian mRNAs. *Elife* **4**, doi:10.7554/eLife.05005 (2015).

4 Lim, L. P. *et al.* Microarray analysis shows that some microRNAs downregulate large numbers of target mRNAs. *Nature* **433**, 769-773, doi:10.1038/nature03315 (2005).

5 Jones, P. A. Functions of DNA methylation: islands, start sites, gene bodies and beyond. *Nat Rev Genet* **13**, 484-492, doi:10.1038/nrg3230 (2012).

6 Hoshida, Y. Nearest template prediction: a single-sample-based flexible class prediction with confidence assessment. *PLoS One* **5**, e15543, doi:10.1371/journal.pone.0015543 (2010).

7 Kumar, L. & M, E. F. Mfuzz: a software package for soft clustering of microarray data. *Bioinformation* **2**, 5-7, doi:10.6026/97320630002005 (2007).

8 Zhou, Y. *et al.* Metascape provides a biologist-oriented resource for the analysis of systems-level datasets. *Nat. Commun.* **10**, 1523, doi:10.1038/s41467-019-09234-6 (2019).

9 Bindea, G. *et al.* Spatiotemporal dynamics of intratumoral immune cells reveal the immune landscape in human cancer. *Immunity* **39**, 782-795, doi:10.1016/j.immuni.2013.10.003 (2013).

10 Janakiram, M., Chinai, J. M., Zhao, A., Sparano, J. A. & Zang, X. HHLA2 and TMIGD2: new immunotherapeutic targets of the B7 and CD28 families. *Oncoimmunology* **4**, e1026534, doi:10.1080/2162402x.2015.1026534 (2015).

11 Wang, J. *et al.* Fibrinogen-like Protein 1 Is a Major Immune Inhibitory Ligand of LAG-3. *Cell* **176**, 334-347.e312, doi:10.1016/j.cell.2018.11.010 (2019).

12 Ward-Kavanagh, L. K., Lin, W. W., Šedý, J. R. & Ware, C. F. The TNF Receptor Superfamily in Co-stimulating and Co-inhibitory Responses. *Immunity* **44**, 1005-1019, doi:10.1016/j.immuni.2016.04.019 (2016).

13 Yoshihara, K. *et al.* Inferring tumour purity and stromal and immune cell admixture from expression data. *Nat. Commun.* **4**, 2612, doi:10.1038/ncomms3612 (2013).

14 Wang, S., He, Z., Wang, X., Li, H. & Liu, X. S. Antigen presentation and tumor immunogenicity in cancer immunotherapy response prediction. *Elife* **8**, doi:10.7554/eLife.49020 (2019).

15 Ayers, M. *et al.* IFN-γ-related mRNA profile predicts clinical response to PD-1 blockade. *J. Clin. Invest.* **127**, 2930-2940, doi:10.1172/jci91190 (2017).

16 Chen, D. S. & Mellman, I. Oncology meets immunology: the cancer-immunity cycle. *Immunity* **39**, 1-10, doi:10.1016/j.immuni.2013.07.012 (2013).

17 Hoshida, Y., Brunet, J. P., Tamayo, P., Golub, T. R. & Mesirov, J. P. Subclass mapping: identifying common subtypes in independent disease data sets. *PLoS One* **2**, e1195, doi:10.1371/journal.pone.0001195 (2007).

18 Nathanson, T. *et al.* Somatic Mutations and Neoepitope Homology in Melanomas Treated with CTLA-4 Blockade. *Cancer Immunol Res* **5**, 84-91, doi:10.1158/2326-6066.Cir-16-0019 (2017).

19 Kim, J. Y., Choi, J. K. & Jung, H. Genome-wide methylation patterns predict clinical benefit of immunotherapy in lung cancer. *Clin Epigenetics* **12**, 119, doi:10.1186/s13148-020-00907-4 (2020).

20 Pusztai, L. *et al.* Durvalumab with olaparib and paclitaxel for high-risk HER2-negative stage II/III breast cancer: Results from the adaptively randomized I-SPY2 trial. *Cancer Cell* **39**, 989-998.e985, doi:10.1016/j.ccell.2021.05.009 (2021).
