# Supplementary material for: Multi-omics analysis of epigenetic dysregulation reveals clinical heterogeneity and evaluates the immunotherapeutic potential of lung adenocarcinoma
Source: Genes Dis. 2025 Feb 20;12(5):101561. doi: 10.1016/j.gendis.2025.101561 (PMC12166690; doi:10.1016/j.gendis.2025.101561)
Supplement: Multimedia component 2 [file mmc2.docx]

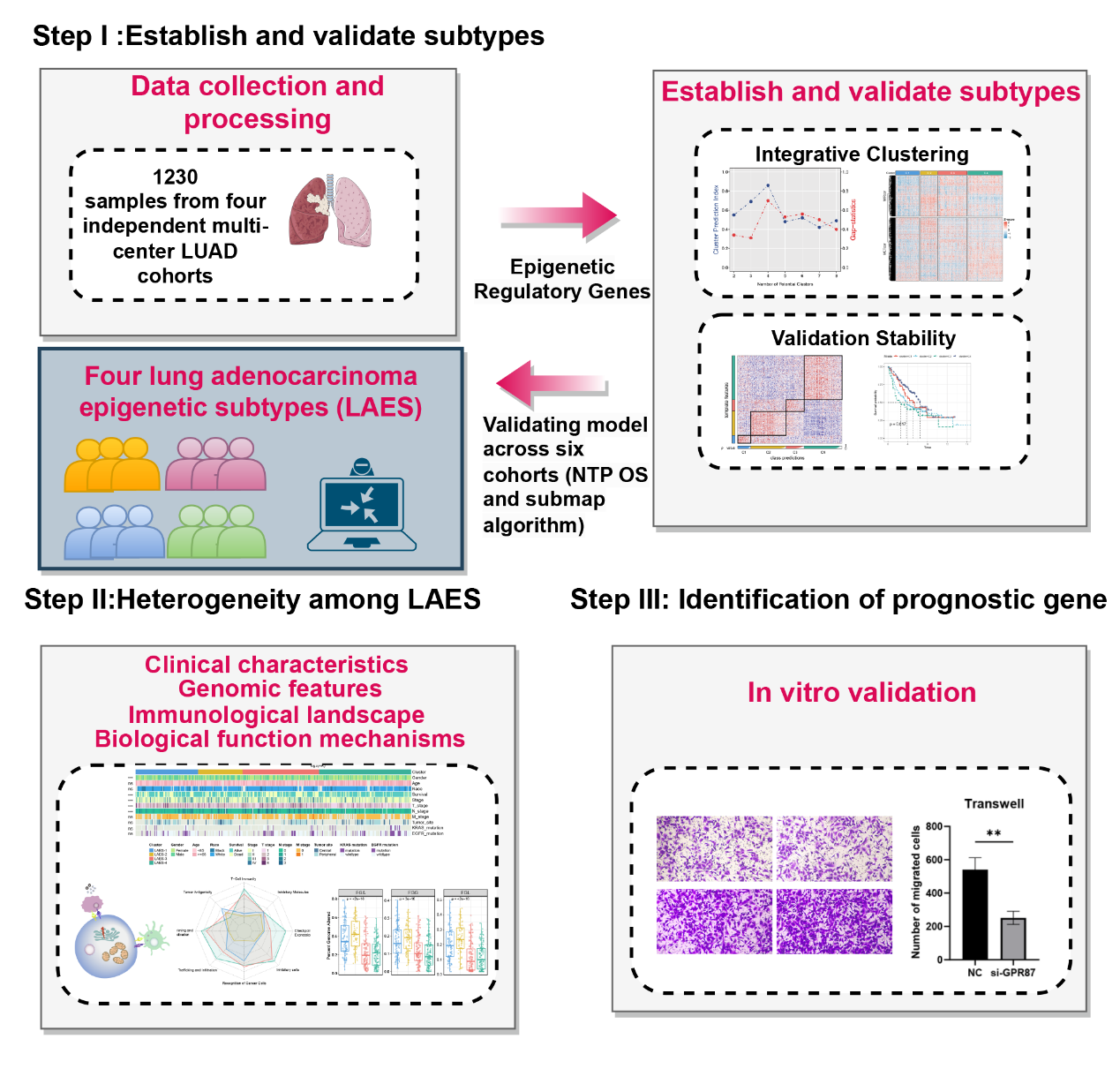


**Fig. S1.** **The flowchart of this study.** The construction of molecular subtypes of LUAD driven by epigenetic regulation-related genes and the analysis of LUAD subtypes in terms of prognosis, functional analysis, clinical features, and further exploration of the immune landscape, immunotherapeutic potential, and multi-omics alterations of LUAD subtypes. Then we identified *GPR87* as a promising predictive and prognostic marker in LUAD, and found *GPR87* silencing reduced cell migration and proliferation ability in phenotypically aggressive LUAD cells. The analyses provide new visions into the heterogeneity of LUAD and optimal therapy decisions for LUAD patients.


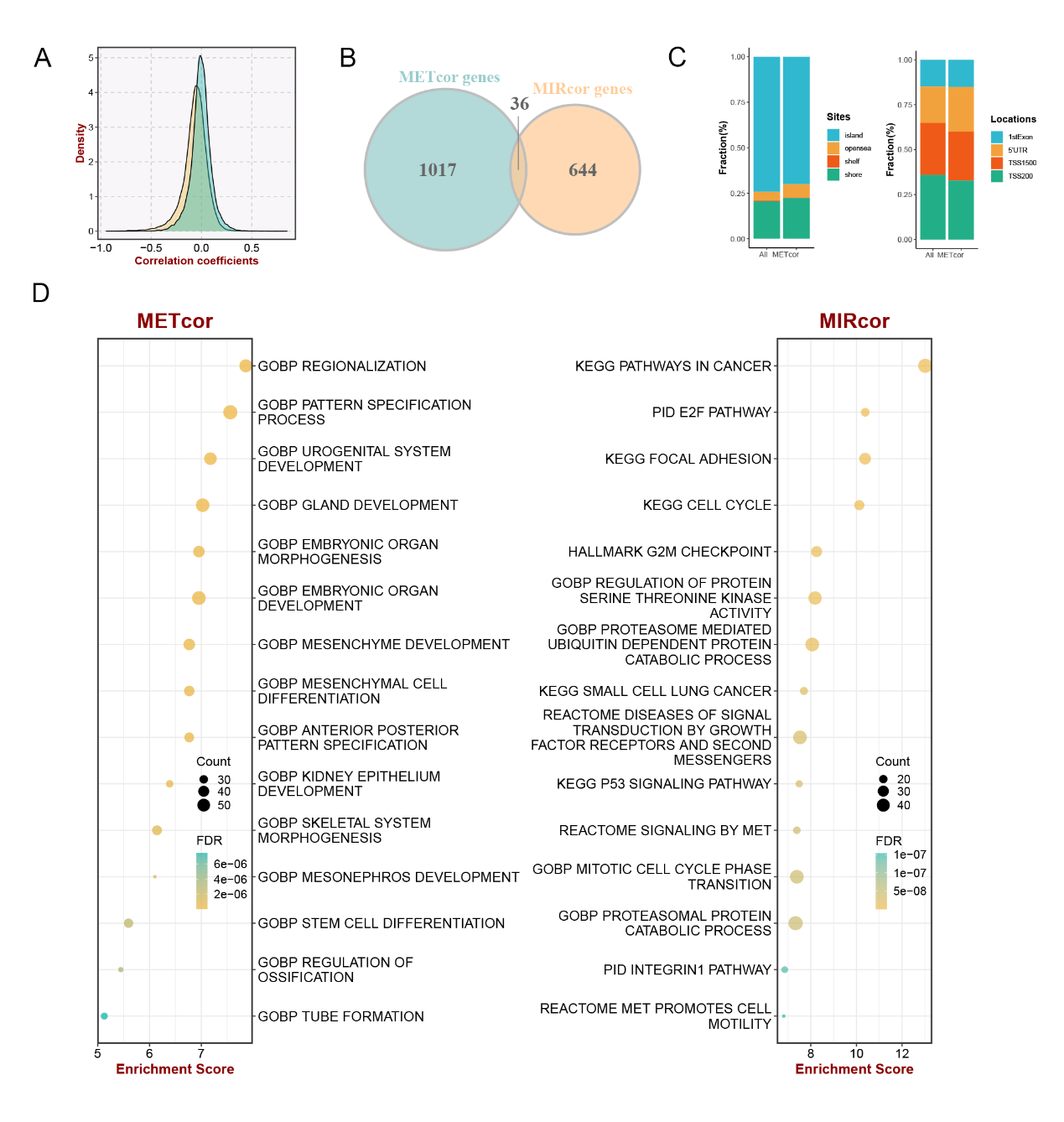


**Fig. S2. Identification of METcor and MIRcor genes and classification of LUAD subtypes. (A)** Distribution of the correlation coefficients between the mRNA expression and DNA methylation or miRNA expression in the TCGA dataset. **(B)** Overlap of the METcor and MIRcor genes. **(C)** The proportional frequencies of promoter CpG sites based on their distance relative to CpG islands and genomic locations. **(D)** Pathway analyses of the METcor and MIRcor genes.


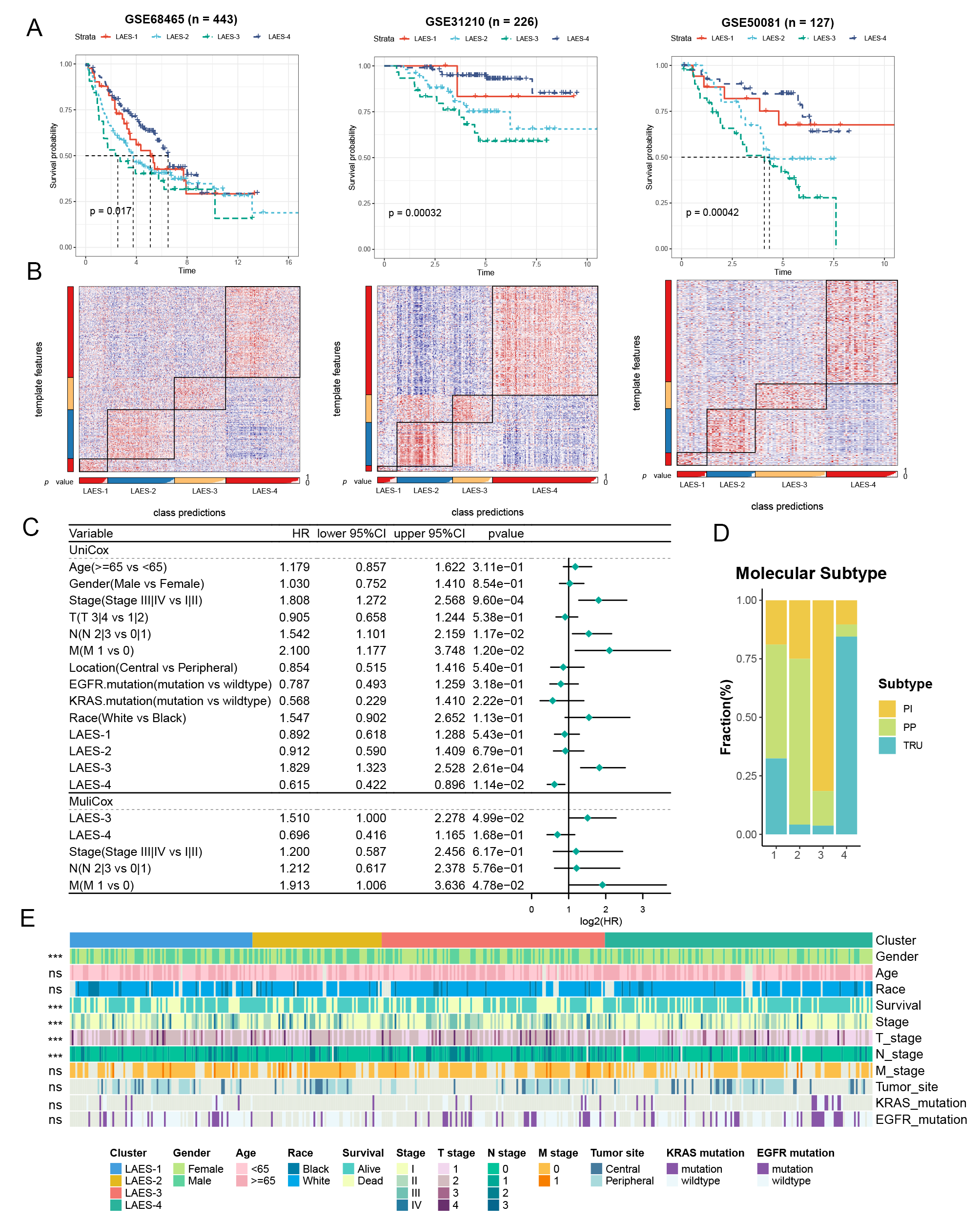


**Fig. S3. Validation of the prognostic significance and clinical features of LAES.** **(A)** Kaplan–Meier survival curves of OS for the LUAD subtypes of three independent datasets (GSE68465: *P* = 0.017; GSE31210: *P* = 0.00032; GSE50081: *P* = 0.00042). **(B)** NTP algorithm predicted the four LUAD subtypes in three independent GEO data sets. **(C)** Graph of clinical features by univariate and multivariate logistic regression analysis. **(D)** Correlation Bar plot showing previously defined molecular types in our LUAD subtypes. **(E)** Bar plot showing previously defined molecular types in our LUAD subtypes. ^ns^*P* > 0.05, **P* < 0.05, ***P* < 0.01, ****P* < 0.001, *****P* < 0.0001.


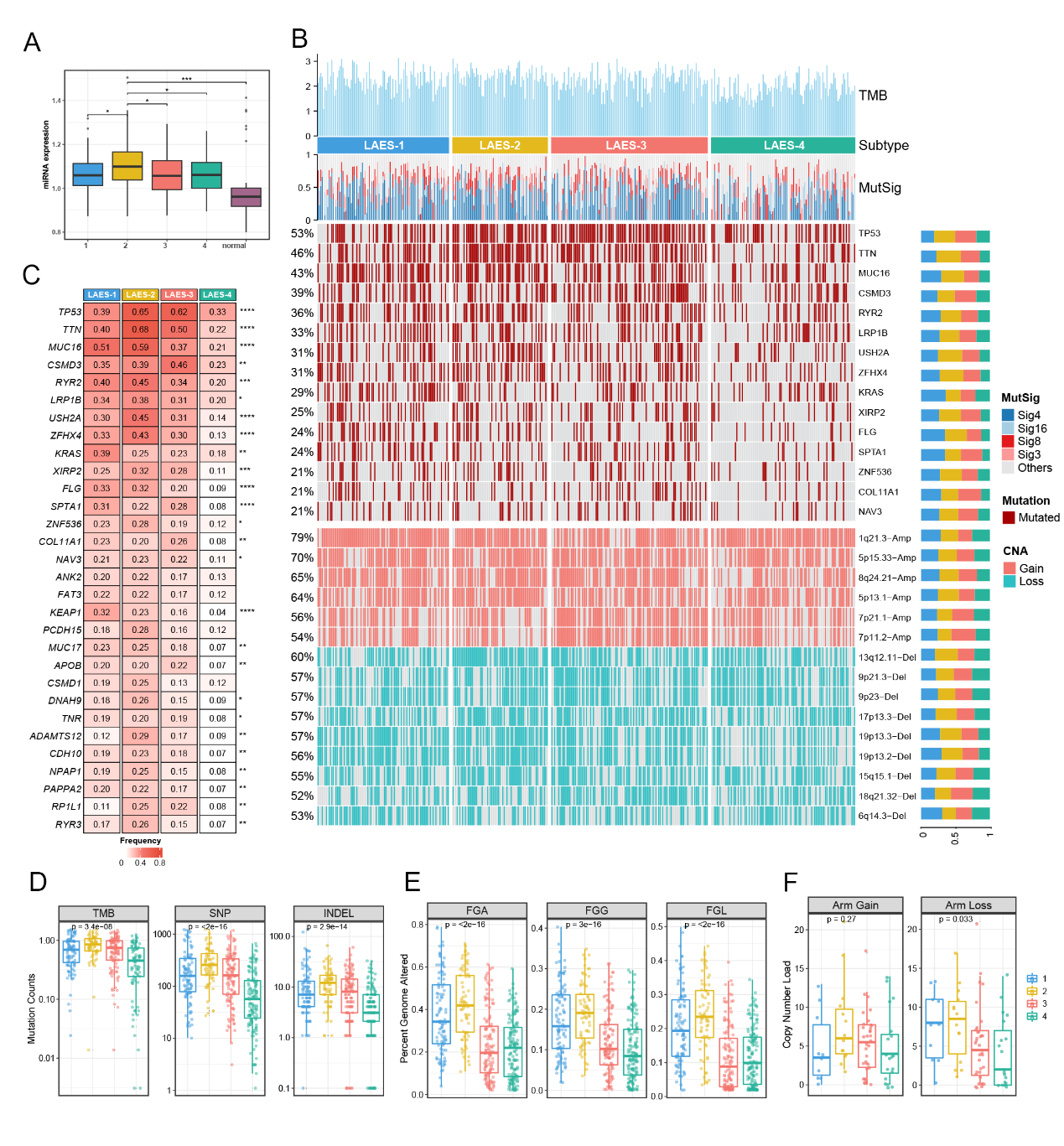


**Fig. S4. Multi-omics landscape of LAES. (A)** The levels of miRNA expression in the LUAD subtypes and adjacent normal samples. **(B)** Genomic alteration landscape of LUAD subtypes. **(C)** The mutation frequency of 30 FMGs among each subtype. **(D)** Distributions of TMB, SNP, and Indel in four subtypes. **(E)** Distributions of FGA, FGG, and FGL among four subtypes. **(F)** Distributions of arm gain, arm loss, focal gain, and focal loss.


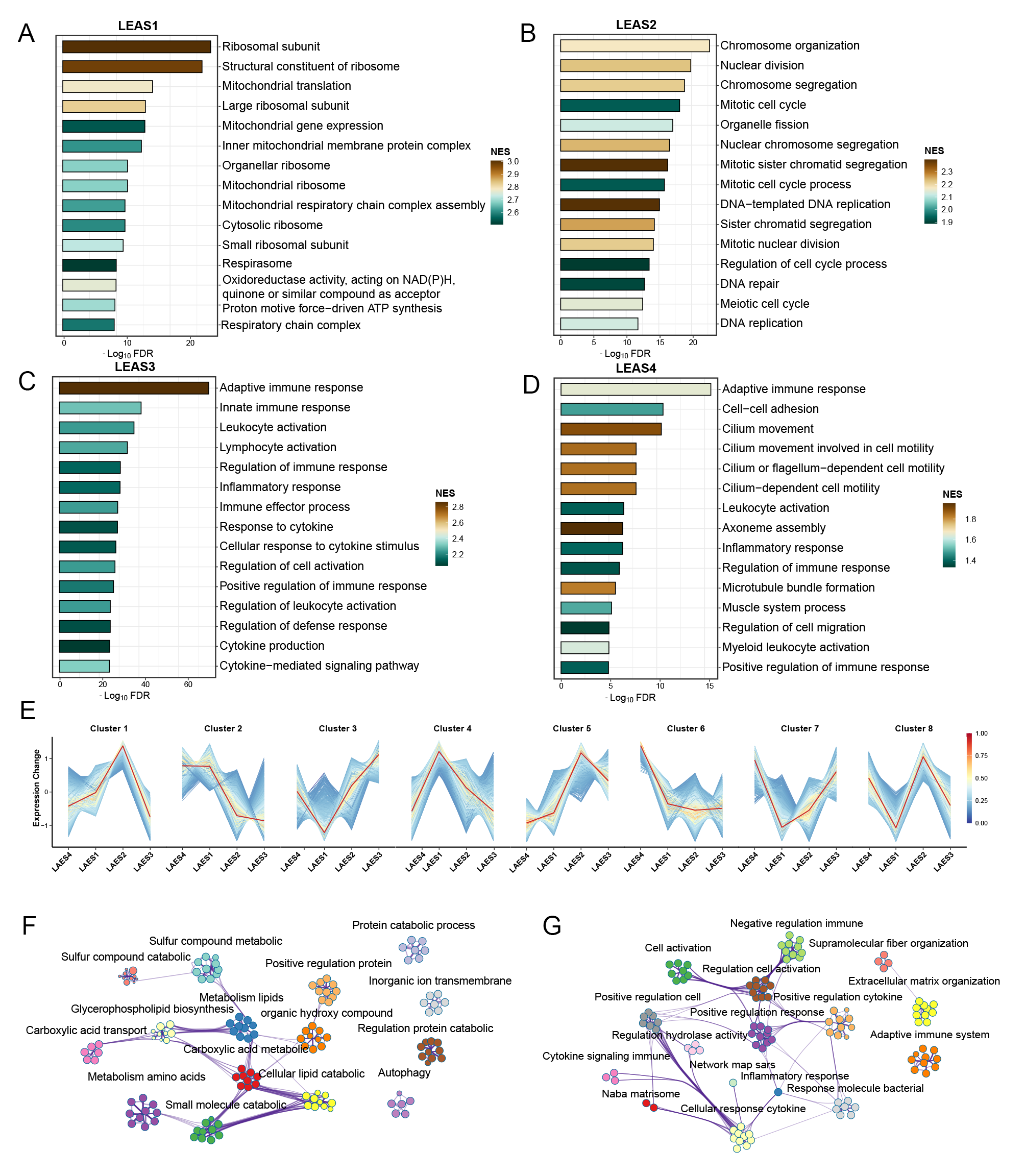


**Fig. S5. Biological processes associated with LAES.** The significantly positive GO terms with ranked differential genes of LAES-1 **(A)**, LAES-2 **(B)**, LAES-3 **(C)** and LAES-4 **(D)**. **(E)** The Mfuzz algorithm identified eight gene clusters in LAES. Heatmaps of biological processes for the LUAD subtypes in TCGA datasets. Metascape functional analysis of feature genes from Cluster 2 **(F)** and Cluster 3 **(G)**.


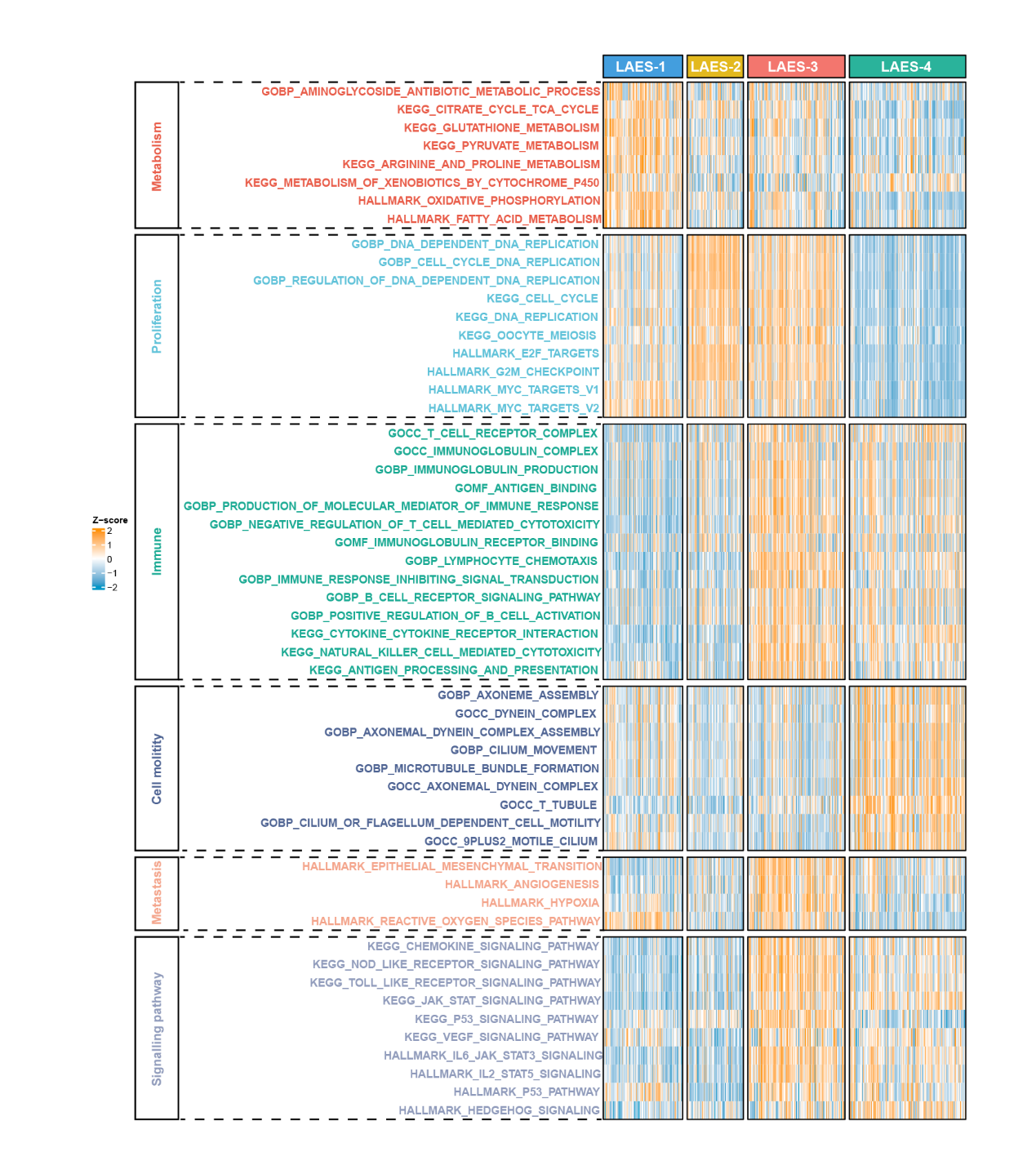


**Fig. S6. Significant biological processes in four LUAD subtype.** Heatmaps of biological processes for the LUAD subtypes in TCGA datasets. High and low ssGESA scores are represented in red and blue, respectively.


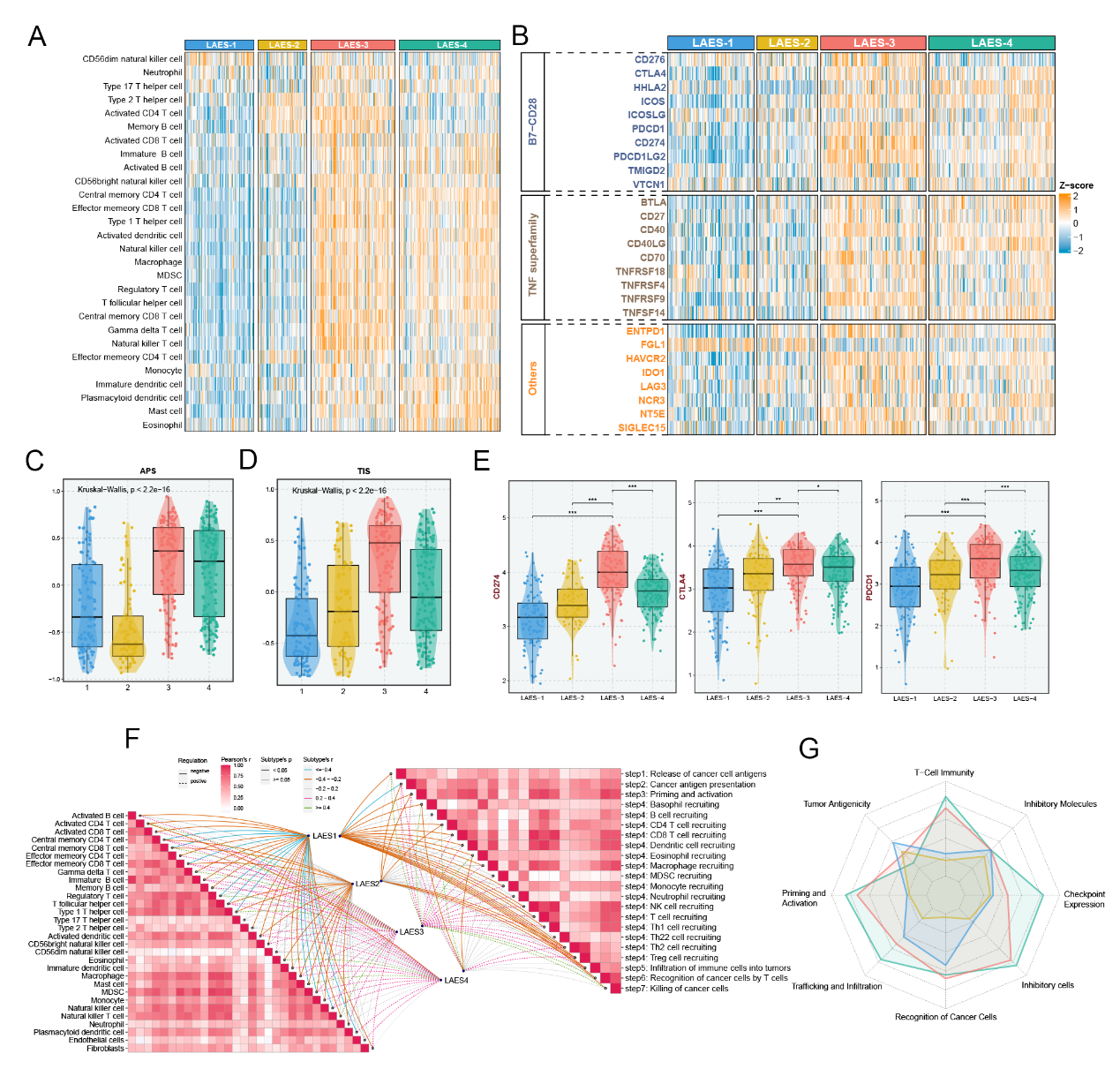


**Fig. S7.** **Immunological characteristics and prediction of immunotherapy.** **(A)** The heatmap of the immune cells infiltration in four subtypes. **(B)** The heatmap of the immune checkpoint molecules expression in LAES. High and low ssGESA scores are represented in red and blue, respectively. **(C)** The relative antigen processing and presenting machinery score is determined for LAES. **(D)** The difference in TIS scores between LAES. **(E)** The relative expression of *CD274*, *CTLA4*, and *PDCD1* was evaluated in the TCGA cohort. **(F)** The correlation of LAES with the steps in CIC and the expression of immune cells. **(G)** The radar chart shows the differences of the CIC immunogram in LAES. ^ns^*P* > 0.05, **P* < 0.05, ***P* < 0.01, ****P* < 0.001, *****P* < 0.0001.


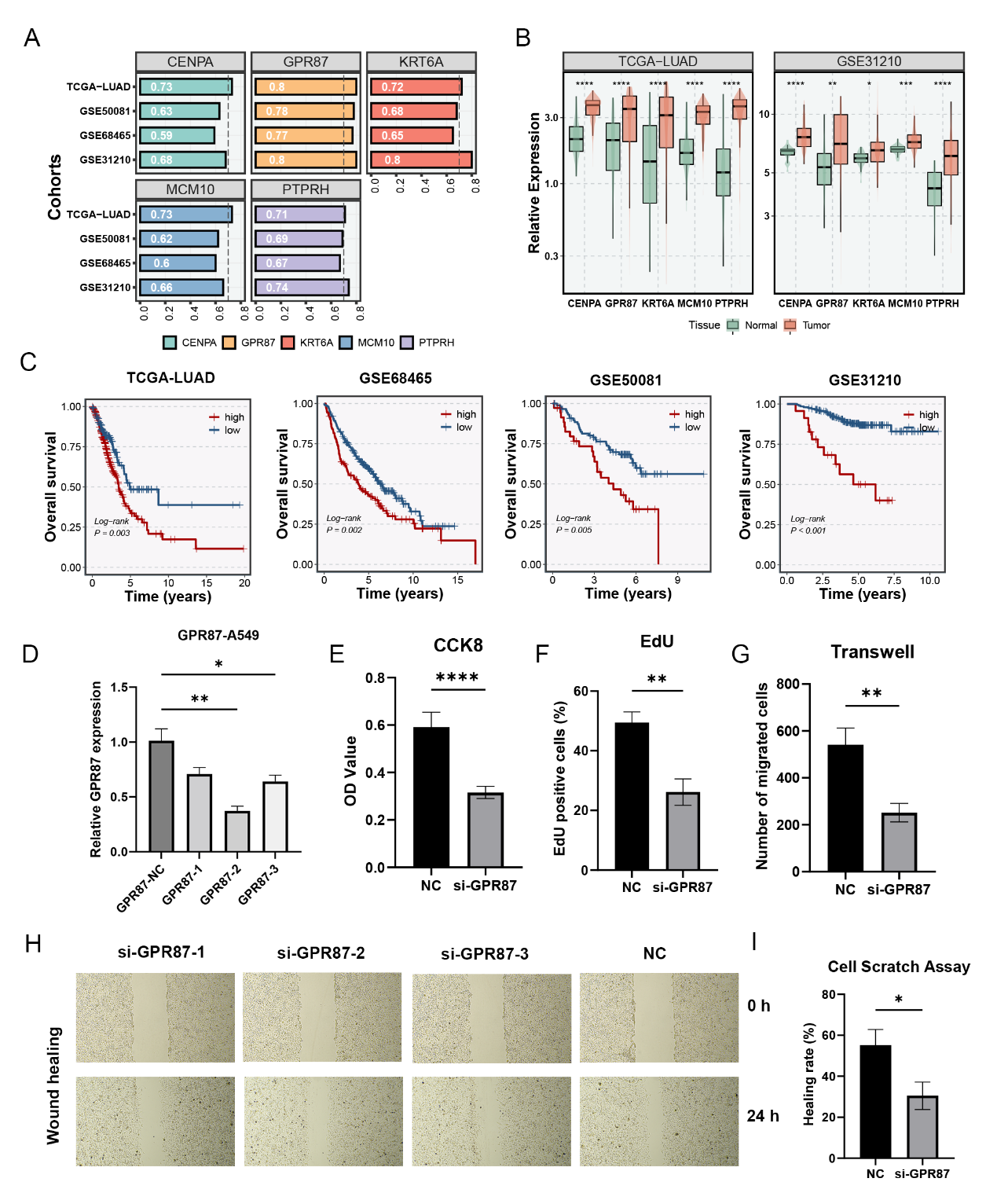


**Fig. S8.** **Identification of prognostic feature *GPR87* in LAES.** **(A)** AUC values of feature genes in all discovery and validation cohorts. **(B)** Differences in gene expression between tumor and normal groups. **(C)** Kaplan-Meier survival curves for the *GPR87*-high and *GPR87*-low groups divided by the best cut-off point in the four discovery and validation cohorts. **(D)** qRT-PCR analysis of the efficiency of si-GPR87 and si-Control (NC) transfection in A549 cells. **(E)** CCK8 proliferation assay was conducted to estimate the function of *GPR87* on A549 cell lines. **(F)** Quantifications of EdU immunofluorescence staining assay in A549 cells. **(G)** Transwell assays were performed to examine the potential migration of A549 cells. **(H-I)** Wound healing assays of cell migration in A549 cells lines or negative control cells at 0, 24h after scratching. ^ns^*P* > 0.05, **P* < 0.05, ***P* < 0.01, ****P* < 0.001, *****P* < 0.0001.
